# Supplementary material for: Proteomic profiling of zinc homeostasis mechanisms in Pseudomonas aeruginosa through data-dependent and data-independent acquisition mass spectrometry
Source: bioRxiv. 2025 Jan 31:2025.01.13.632865. Originally published 2025 Jan 13. Preprint. [Version 2] doi: 10.1101/2025.01.13.632865 (PMC11761036; doi:10.1101/2025.01.13.632865)

***Pseudomonas aeruginosa* PAO1 Cellular Overview**

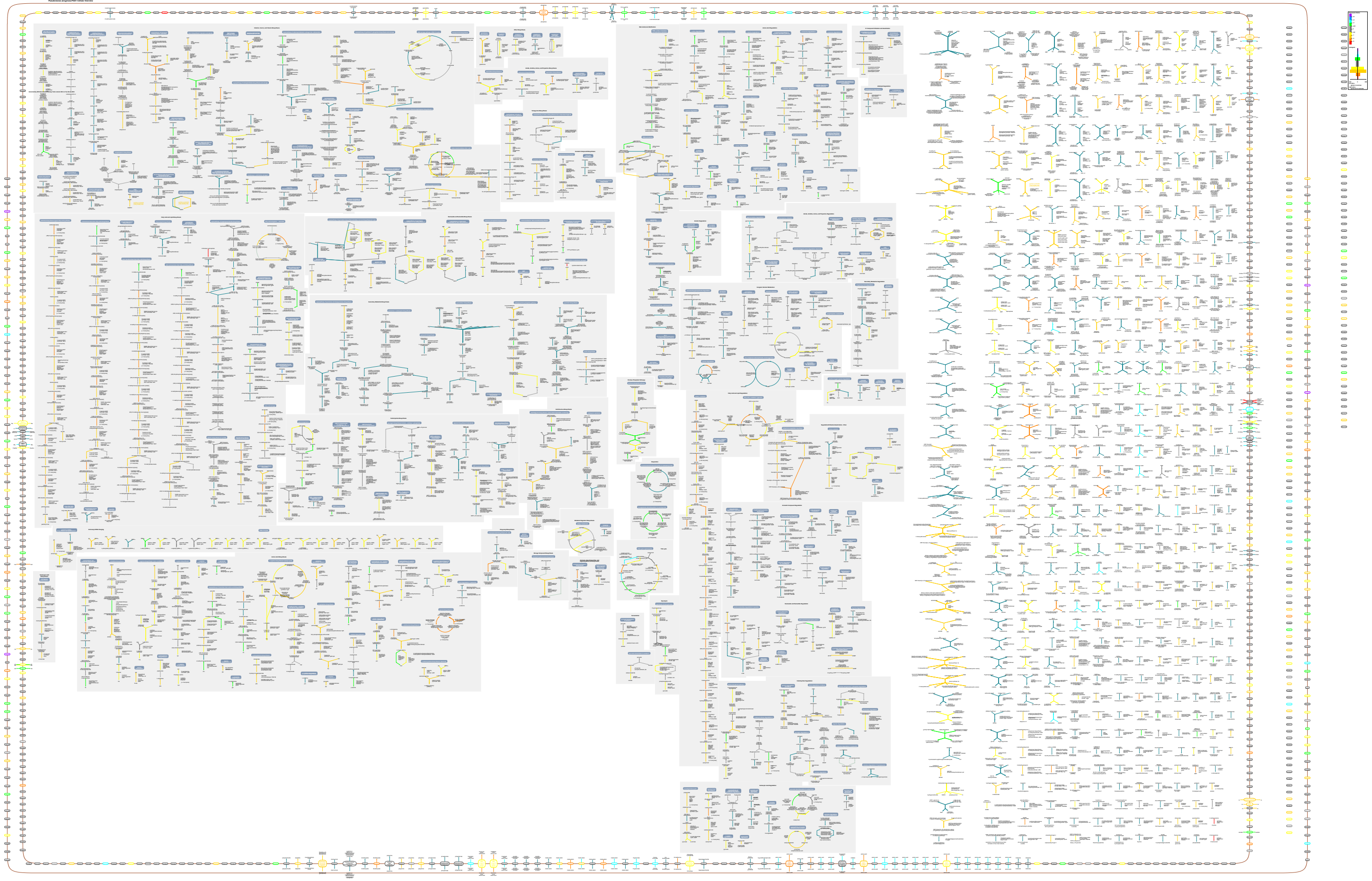

Supplement: Supplement 5 [file media-5.pdf]
