# Supplementary material for: Proteomic profiling of zinc homeostasis mechanisms in Pseudomonas aeruginosa through data-dependent and data-independent acquisition mass spectrometry
Source: bioRxiv. 2025 Jan 13:2025.01.13.632865. Preprint. [Version 1] doi: 10.1101/2025.01.13.632865 (PMC11761036; doi:10.1101/2025.01.13.632865)
Supplement: Supplementary file 1 [file NIHPP2025.01.13.632865V1-supplement-1.pdf]

bioRxiv preprint doi: https://doi.org/10.1101/2025.01.13.632865; this version posted January 13, 2025. The copyright holder for this preprint (which was not certified by peer review) is the author/funder, who has granted bioRxiv a license to display the preprint in perpetuity. It is made available under aCC-BY 4.0 International license.

Supplemental

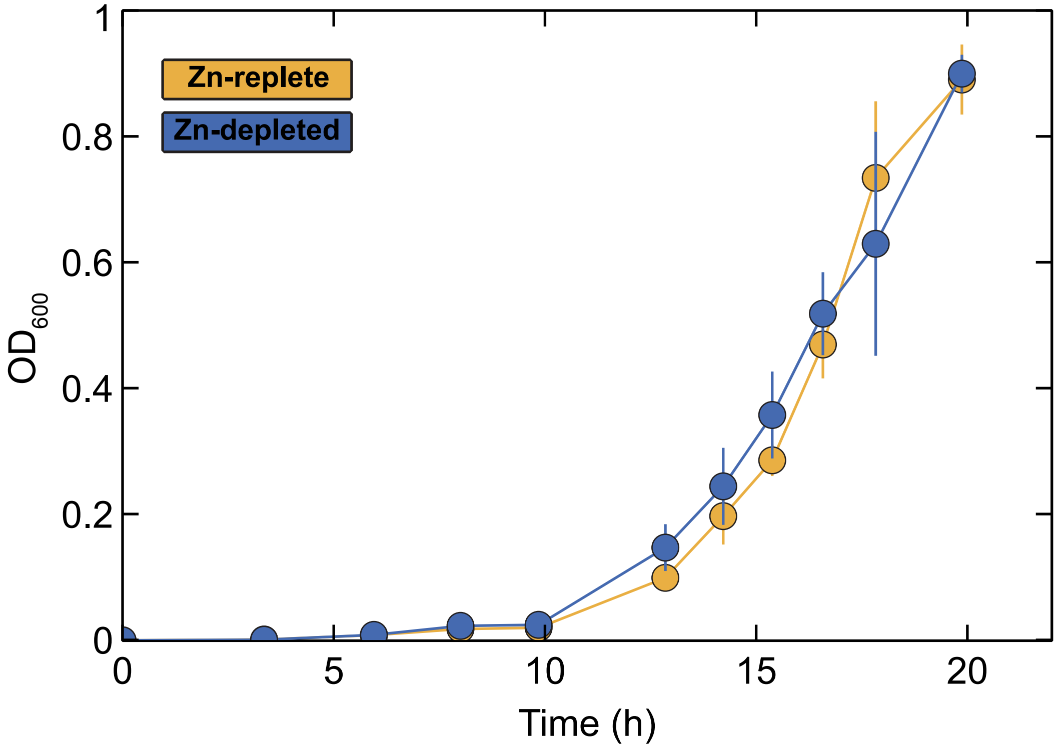

Figure S1. Growth curve of *P. aeruginosa* samples grown in Zn-replete (yellow) and Zn-depleted (blue) conditions, as determined by OD_{600} measurements. Error bars represent the standard deviation of biological triplicate samples.
